# Overexpression of Human *SNX27* Enhances Learning and Memory Through Modulating Synaptic Plasticity in Mice

**DOI:** 10.3389/fcell.2020.595357

**Published:** 2020-11-27

**Authors:** Yuanhui Huo, Yue Gao, Qiuyang Zheng, Dongdong Zhao, Tiantian Guo, Shuo Zhang, Yuzhe Zeng, Yiyun Cheng, Huaping Gu, Lishan Zhang, Bin Zhu, Hong Luo, Xian Zhang, Ying Zhou, Yun-wu Zhang, Hao Sun, Huaxi Xu, Xin Wang

**Affiliations:** ^1^State Key Laboratory of Cellular Stress Biology, Fujian Provincial Key Laboratory of Neurodegenerative Disease and Aging Research, Institute of Neuroscience, School of Medicine, Xiamen University, Xiamen, China; ^2^National Institute for Data Science in Health and Medicine, School of Medicine, Xiamen University, Xiamen, China

**Keywords:** SNX27, trafficking, synaptic plasticity, learning and memory, glutamate receptors

## Abstract

Abnormal synaptic transmission leads to learning and memory disorders and is the main feature of neurological diseases. Sorting nexin 27 (SNX27) is an endosomal adaptor protein associated with a variety of nervous system diseases, and it is mainly responsible for the trafficking of postsynaptic membrane receptors. However, the roles of SNX27 in regulating synaptic and cognitive function are not fully understood. Here, we first generated a neuron-specific human-*SNX27* transgenic mouse model (h*SNX27* Tg) that exhibited enhanced excitatory synaptic transmission and long-term potentiation (LTP). In addition, we found that the h*SNX27* Tg mice displayed enhanced learning and memory, lower-level anxiety-like behavior, and increased social interaction. Furthermore, we found that SNX27 overexpression upregulated the expression of glutamate receptors in the cortex and hippocampus of h*SNX27* Tg mice. Together, these results indicate that SNX27 overexpression promotes synaptic function and cognition through modulating glutamate receptors.

## Introduction

Synaptic function, especially the function of excitatory synapses, is closely associated with learning and memory (Kasai et al., 2010). Glutamate acts as the main neurotransmitter of excitatory neurons in mammalian brains and plays a key role in cognitive function (Niciu et al., 2012). Ionotropic α-Amino-3-hydroxy-5-methyl-4-isoxazolepropionic acid (AMPA) and *N*-methyl-D-aspartate (NMDA) glutamate receptors are two major types of postsynaptic receptors in glutamatergic synapses, which transmit presynaptic signals to postsynaptic neurons (Sheng and Kim, 2011). Activation of NMDA receptors leads to long-term potentiation (LTP), which is regarded as a way of encoding “memories” in mammalian brains (Malenka, 2003; Kessels and Malinow, 2009). Mutations or abnormal expression of GluN1 (Chen et al., 2017), GluN2B (Hu et al., 2016) and GluA1 (Geisheker et al., 2017) are associated with intellectual disability.

As a member of the sorting nexin family proteins, Sorting nexin 27 (SNX27) contains a conserved phosphoinositide-binding domain (PX domain) which anchors SNX27 to the endosome membrane (Worby and Dixon, 2002; Seet and Hong, 2006; Cullen, 2008). In addition to PX domain, SNX27 contains a PDZ domain that binds transmembrane cargoes with PDZ binding motifs, and a FERM-like domain. Multiple transmembrane cargo proteins, including ion channels and receptors, are transported to plasma membrane by the SNX27-retromer complex (Gallon et al., 2014; Schulz and Hausmann, 2016).

Given that synaptic dysfunction and endo-lysosomal dysregulation represent the pathological features in neurodegenerative diseases (Acquarone et al., 2019; Chakroborty et al., 2019; Van Acker et al., 2019), such as Alzheimer’s disease (AD) and Down’s syndrome (DS), the role of SNX27 in these diseases has attracted extensive attention. In our previous study, we found premature lethality in *Snx27*^–/–^ mice. Moreover, we found that *Snx27*^+/–^ mice featured synaptic dysfunction and cognitive impairment, as well as the reduction in postsynaptic AMPA and NMDA receptors compared with that in *Snx27*^+/+^ mice (Wang et al., 2013). In addition, SNX27 has been found to regulate amyloid precursor protein (APP) processing and Aβ generation through interacting with presenilin 1 (Wang et al., 2014). Furthermore, a deleterious homozygous mutation in SNX27 was identified in a consanguineous family with myoclonic epilepsy and neurodegeneration (Damseh et al., 2015). SNX27 deficiency lead to development and aging-related disorders; however, the physiological and pathological roles of SNX27 overexpression remain largely unclear.

Here, we generated a SNX27 transgenic mouse model that overexpresses human *SNX27* specifically in excitatory neurons under the control of a Calcium/Calmodulin Dependent Protein Kinase II Alpha (*Camk2a*) promoter. The human-*SNX27* transgenic (h*SNX27* Tg) mice exhibited enhanced learning and memory, reduced anxiety-like behaviors, and increased social interaction, without seizure-like behavior. Furthermore, SNX27 overexpression enhanced excitatory synaptic transmission and plasticity through upregulating synaptic glutamate receptors and promoting synaptogenesis. These results indicate that SNX27 overexpression promotes synaptic transmission and cognitive function through regulating glutamate receptors in central nervous system.

## Materials and Methods

### Animals

Human-*SNX27* Tg mice overexpressing human *SNX27* fused with HA under the control of the mouse *Camk2a* promoter were used in this study and were maintained on a C57BL/6 background. A piggyBac transposon gene expression vector and helper plasmid were injected into C57BL/6 embryos to insert a segment containing the 1,296 bp mouse *Camk2a* promoter and the 1,614 bp human *SNX27* sequence with HA tag and thereby generate h*SNX27* Tg mice. The offspring mice were genotyped by PCR primers: F1: 5′-GATGAGATGCAGCGATGGG ACAC-3′, R1: 5′-AGCCAGAAGTCAGATGCTCAAGG-3′, F2: 5′-CAACCACT TACAAGAGACCCGTA-3′, R2: 5′-GAGCCC TTAGAAATAACGTTCACC-3′.

Analysis was performed using 1% agarose gel. A 632 bp product was verified for the wild-type (WT) allele, and a 368 bp product was amplified for the transgenic allele.

Age-matched WT littermate mice were used as controls. All animal experiments were conducted in strict accordance with the guidelines of the Institutional Animal Care and Use Committee of Xiamen University.

### Quantitative Real-Time PCR

Total RNA was extracted from brain tissues using TRIzol reagent (Thermo Fisher Scientific, Waltham, MA, United States) according to the manufacturer’s instructions. cDNA was reverse transcribed using the ReverTra Ace qPCR RT Kit (TOYOBO, Osaka, Japan). Quantitative real-time PCR (qRT-PCR) was accomplished using 7500 Fast V2.0.6 (Invitrogen, Shanghai, China) with the SYBR^®^ Prime Script miRNA RT-PCR Kit (Takara). *Actb* was used as an internal control for quantitative analysis using the 2^–ΔΔ*Ct*^ method. The gene primers used for real-time PCR were as follows: Mouse *Snx27*: 5′-GTGAACGGCGTGAATGTTGAG-3′ and 5′-ACTGGGATCTAGGTTATC AGCC-3′; Mouse *Grin1*: 5′-A GCCCAACGCCATACAGATG-3′ and 5′-ACGCGCATCATCT CAAACCA-3′; Mouse *Grin2a*: 5′-TGTGAACGTGGTGGC GTTAT-3′ and 5′-TGGAAGAACGTGGATGTCGG-3′; Mouse *Grin2b*: 5′-ACGTCTCAAACC CTCGACAC-3′ and 5′-GCG GCTCACAATGCAGAATC-3′; Mouse *Gria1*: 5′-CGG TTTTCT AGGTGCGGTTG-3′ and 5′-CCAAAGATGGCGTACACTCCT-3′; Mouse *Gria2*: 5′-GGGGACTGATTTTTGGTGTCTC-3′ and 5′-GCCTCTTGAAAACTGGG AGC-3′; and *Actb*: 5′-AGCCATGTACGTAGCCATCCA-3′ and 5′-TCTCCGGAGT CCATCACAATG-3′.

### Western Blot Analysis

Brain tissues were lysed in RIPA buffer (150 mM NaCl, 25 mM Tris–HCl, pH 7.5, 1% Nonidet P-40, 0.1% sodium dodecyl sulfate) supplemented with protease inhibitor cocktail (Roche, Mannheim, Germany). Protein lysates were denatured with SDS protein loading buffer, subjected to polyacrylamide gel electrophoresis (SDS-PAGE), and then transferred to polyvinylidene difluoride (PVDF) membranes (Millipore, Billerica, MA, United States). The membranes containing the extracted proteins were blocked with 5% skim milk and then incubated with primary antibodies and horseradish peroxidase (HRP)-conjugated secondary antibodies (31430 or 31460, Thermo Fisher Scientific, Waltham, MA, United States). Finally, protein band intensity was quantified using ImageJ software (National Institutes of Health, Bethesda, MD, United States) (Zheng et al., 2017).

The following primary antibodies (all diluted 1:1,000) were used: rabbit anti-SNX27 (derived as described previously) (Cai et al., 2011), mouse anti-HA (901503, BioLegend, San Diego, CA, United States), rabbit anti-β-actin (Cell Signaling Technology, Danvers, MA, United States), rabbit anti-GluN2A (MAB5216, Millipore), mouse anti-GluN2B (610416, Millipore), rabbit anti-GluA1 (04-855, Millipore), mouse anti-GluA2 (AB1768, Millipore), mouse anti-GluN1 (MAB397, BD Biosciences, San Jose, CA, United States), mouse anti-synaptophysin (SAB4200544, Sigma, Burlington, MA, United States), mouse anti-γ-aminobutyric acid type A receptor β3 subunit (GABA_*A*_Rβ3) (sc-376252, Santa Cruz Biotechnology, Dallas, TX, United States), mouse anti-glutamic acid decarboxylase 65-kilodalton isoform (GAD-65) (sc-377145, Santa Cruz Biotechnology) and mouse anti-PSD95 (MAB1598, Millipore).

### Immunohistochemistry

Mouse brains were perfused with 4% paraformaldehyde (PFA) followed by dehydration with 30% sucrose. Then, 30-μm sections were obtained using a freezing microtome (CM1950, Leica Buffalo Grove, United States) and rinsed with PBS. After high-temperature retrieval in sodium citrate buffer (MVS-0100, MX Biotechnologies) for 15 min, the sections were incubated with primary antibodies overnight at 4°C and Alexa Fluor-conjugated secondary antibodies (Invitrogen, Carlsbad, CA, United States) for 2 h and then sealed with glycerin. Images were captured by confocal fluorescence microscope (Olympus, Japan). The antibodies used were as follows: rabbit anti-SNX27 (derived as described previously) (Cai et al., 2011); mouse anti-NeuN (MAB377, Millipore) and goat anti-mouse Alexa Fluor 405 (A-31553)/488 (A-21141)/594 (A-21145) IgG (H + L) and goat anti-rabbit Alexa Fluor 405 (A-31556)/488 (A32731)/594 (A32740) IgG (H + L), both from Thermo Fisher Scientific.

### Nissl Staining

Mouse brain sections were stained using the Nissl Staining Kit (Beyotime, Shanghai, China) according to the manufacturer’s protocol. Images were collected by optical microscopy and analyzed by ImageJ software.

### Golgi Staining and Analysis

Brain slices were stained using the FD Rapid Golgi Stain Kit (FD NeuroTechnologies, Columbia, MD, United States) according to the manufacturer’s instructions. Images were obtained using confocal microscope with Z-stack-compression and imported into ImageJ for calculating spine density.

### EEG Recording

Human-*SNX27* Tg and WT littermates were subjected to electroencephalogram (EEG) recording as previously described (Buckmaster and Lew, 2011; Zheng et al., 2016). C57BL/6 mice injected with Kainic acid (KA) (15 mg/kg, *i.p.*) were used as positive controls. In brief, mice were anesthetized with isoflurane (0.5%) and kept warm throughout the experiment. A 0.1 mm diameter insulated microelectrode (Plastics One Inc., Roanoke, VA, United States) was implanted into the frontal and temporal area of each hemisphere (mediolateral, ±1.8 mm, anteroposterior, −1.5 mm from the bregma). Another insulated stainless-steel wire (50 μm diameter, California Fine Wire) was inserted 1.7 mm under the mouse scalp. In addition, a reference electrode was planted in the cerebellum. Data were acquired and analyzed by Nicolet software (version 1.0).

### Electrophysiology

Electrophysiology was performed as previously described (Zeng et al., 2019). The 400-μm mouse brain slices were prepared using a Leica VT1200S vibrating microtome in ice-cold dissection solution (64 mM NaCl, 2.5 mM KCl, 1.25 mM NaH_2_PO_4_, 10 mM MgSO_4_, 0.5 mM CaCl_2_, 26 mM NaHCO_3_, 10 mM D-glucose, 120 mM sucrose, pH 7.4, 290–320 mOsm). Slices were left undisturbed for 1 h at 32°C and then equilibrated at room temperature for 1 h in artificial cerebrospinal fluid (aCSF) containing 126 mM NaCl, 3.5 mM KCl, 1.25 mM NaH_2_PO_4_, 1.3 mM MgSO_4_, 2.5 mM CaCl_2_, 11 mM NaHCO_3_, and 10 mM glucose, pH 7.4, 290–300 mOsm. Slices were transferred to the recording chamber and perfused with aCSF (2 ml/min). All recordings were performed at room temperature. All solutions were saturated with 95% O_2_/5% CO_2_ (v/v).

For LTP recoding, field excitatory postsynaptic potentials (fEPSPs) were evoked by stimulating the Schaffer collateral/commissural pathway (for CA1) with a bipolar tungsten electrical stimulating electrode. Based on the stimulus-response curve, a stimulation intensity that evoked a fEPSP response with 30% of the maximum response was selected and was recorded for 20 min (baseline). LTP was induced by high-frequency stimulation (two trains of 100 Hz with 1 s duration and 30 s interval). The field potential response after tetanic stimulation was recorded for 60 min. LTP magnitude was quantified as the percentage change in the average fEPSP slope over 60 min after induction.

For whole-cell patch-clamp recording, all experiments were conducted on CA1 pyramidal neurons. Miniature excitatory postsynaptic current (mEPSC) and miniature inhibitory postsynaptic current (mIPSC) were recorded at a holding potential of −70 and 0 mV, respectively. Glass pipettes were filled with solution containing 140 mM CsCH_3_SO_3_, 2 mM MgCl_2_⋅6H_2_O, 5 mM TEA-CL, 10 mM HEPES, 1 mM EGTA, 2.5 mM Mg-ATP, and 0.3 mM Na-GTP, pH 7.3, 280 mOsm. Tetrodotoxin at 1 μM was included in the perfusion solution for mEPSC and mIPSC recording. For evoked excitatory postsynaptic current (eEPSC) recording, we obtained input-output relationships at stimulation intensities from 40 to 320 μA. For the paired-pulse experiment, two pulses were measured with various inter-pulse intervals (20, 50, 100, 200, and 500 ms). The paired-pulse ratio was calculated by dividing the eEPSC response to the second pulse by the response to the first pulse. The resistance of the pipettes was 5–8 MΩ. Data were filtered at 2 kHz and sampled at 10 kHz.

### Behavioral Experiments

All experiments were designed to avoid times of rest for the mice. Mice were allowed to adapt to the environment 1 h before testing and subjected to appropriate temperature and light throughout the process. All instruments were cleaned using 75% ethanol.

### Open Field Test

An open field test (OFT) was used to assess athletic ability and anxiety-like responses during the exploratory behavior of mice. Mice were individually placed in the center of an open field (40 × 40 × 40 cm) and allowed to explore freely for 10 min. Total distance, time spent in the center and entry times were recorded and analyzed using the Smart 3.0 video tracking system (Panlab, Harvard Apparatus, Holliston, MA, United States).

### Elevated Plus Maze

An elevated plus maze (EPM) apparatus was used, which included two open arms and closed arms in staggered form. Mice were tested individually, with each mouse placed in the center square facing one of the open arms and away from the researcher. Total distance, duration of time and number of entries into each of the four arms over 8 min of exploration were measured.

### Light/Dark Transition Test

This test was conducted to assess anxiety-like behavior in the mice. The testing box (41 × 41 × 30 cm) consisted of a light chamber and a dark chamber of equal size separated by a partition, through which the test mouse can pass freely. The test mouse was placed in the dark side and then tested for 8 min. The total number of transitions and the total time spent in each chamber were recorded.

### Morris Water Maze Test

The Morris water maze (MWM) was used to analyze reference spatial memory and was performed in a circular pool (diameter 120 cm) filled with water at 22°C, using a modified protocol (Zhao et al., 2018). Around the pool, recognizable and contrasting shapes were provided as reference cues, and a transparent platform (diameter 10 cm) was submerged 1–2 cm under the water. In each trial, a mouse was placed into the water facing the wall of the pool at one of four points (N, S, E, and W) of the maze, selected at random. The mouse was allowed to find the platform for escape; if it had not found the platform after 60 s, it was guided to the hidden platform. During the 4 days training, two trials were conducted daily separated by a 1 h interval, and the latency to reach the hidden platform (escape latency) was scored. In the probe test on day 5, the hidden platform was removed, and the time spent in each quadrant and the number of crossings over the original platform area were analyzed.

### Fear Conditioning Test

A fear conditioning test was performed as previously described (Lee et al., 2017). This test was performed in a soundproof box (22 × 22 × 30 cm) with a metal grid floor capable of delivering an electric shock and a dim light installed in the ceiling. On the first day, the training day, each mouse was allowed to explore the chamber for 120 s to determine the basal freezing response. Then, the mouse was exposed to a tone (80 dB) for 30 s as a conditioning stimulus (CS) and a foot shock (0.5 mA) in the last 2 s as an unconditioned stimulus (US). Three CS-US trials were performed at 30 s intervals, and then the mouse was left undisturbed for another 1 min. In the contextual test, each mouse was placed into the box without any stimulus 24 h after the training period, and freezing behavior was recorded for 5 min. A cued test was also performed, in which each mouse was placed in the box and allowed to explore freely for 3 min, followed by another 3 min in the presence of the CS. Subsequent tests were performed on day 3 and 6 after training to examine memory extinction. Animal freezing responses were recorded and analyzed by using the software (Panlab, Harvard Apparatus, Holliston, MA, United States).

### Novel Object Recognition Test

The novel object recognition test, a non-force driving and spontaneous memory test, was performed. Before training, each mouse was allowed to habituate to the test space (40 × 40 × 40 cm) for 10 min. During the training phase, the mouse was placed in the space with two identical objects, one in each of two corners, and allotted 10 min for exploration. 1 day later, the mouse was tested using the same procedure except that one object had been replaced with a novel object; the mouse was allowed to explore for 10 min. Exploration time was considered to have started when the front paws or nose touch the objects and was analyzed as the discrimination index (DI), DI = T_*novel*_/(T_*novel*_ + T_*familiar*_).

### Object Location Memory Test

During this test, each mouse was allowed to freely explore the two objects in the training stage for 10 min. 2 h later, one object was moved to a new position in the area, and the mouse was allowed to explore for 10 min. Exploration time was recorded as an indicator of spatial memory.

### Statistical Analysis

Unpaired t tests were performed using GraphPad Prism software (GraphPad Software, San Diego, CA, United States). All data represent means ± SEM. Statistical significance was evaluated at *P* < 0.05.

## Results

### Generation of Human-*SNX27* Transgenic Mice

**FIGURE 1:**
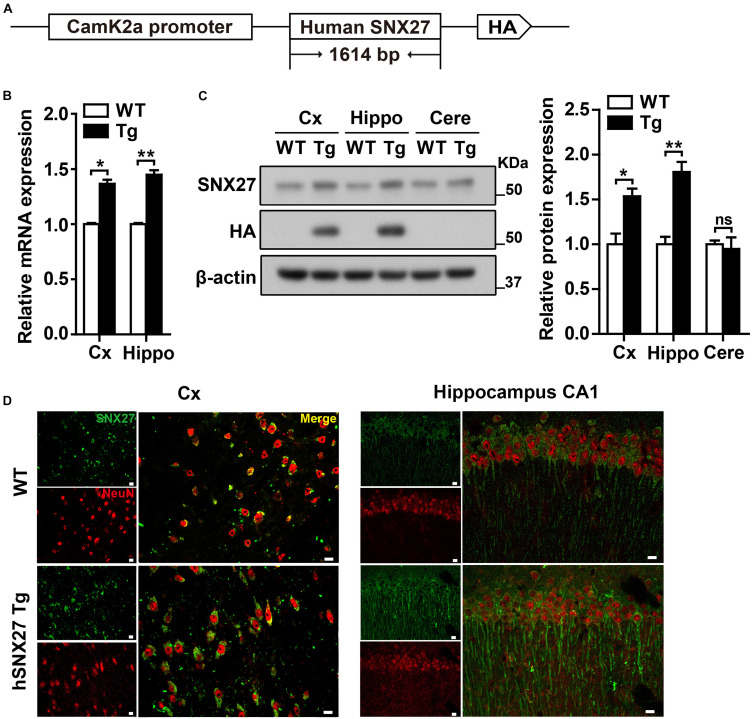
Generation and characterization of human *SNX27* (h*SNX27*) transgenic mice. **(A)** Schematic representation of the h*SNX27* transgenic mouse. **(B)** *SNX27* mRNA levels in WT and Tg mouse brains (Cx, cortex; Hippo, hippocampus) were determined by qRT-PCR; values were normalized to *Actb*. WT values were set as one arbitrary unit. Data represent mean ± s.e.m. (*n* = 3). **(C)** Western blot analysis was conducted to determine SNX27 protein levels in cortex (Cx), hippocampus (Hippo), and cerebellum (Cere) of Tg mice. Lysates from WT were used as negative controls. **(D)** Brain slides from 2-month-old WT and Tg mice were immunostained with SNX27 (in green) and NeuN (in red) and observed by confocal microscopy. Scale bar = 10 μm. ns, not significant, *P < 0.05, **P < 0.01 (two-tailed unpaired *t* test).

We first found that endogenous SNX27 was highly expressed in mouse cortex, hippocampus and cerebellum (Supplementary Figure 1A), and was mainly expressed in neurons and astrocytes, but with a relatively low expression in microglia (Supplementary Figure 1B). To further investigate the function of SNX27 in the central nervous system, we generated an *SNX27* transgenic mouse model (h*SNX27* Tg) that overexpresses human *SNX27* with a HA tag under the control of the mouse *Camk2a* promoter (Figure 1A). Successful generation of h*SNX27* Tg mice was confirmed by PCR-based genotyping (Supplementary Figure 1C). Moreover, we examined the mRNA and protein levels of SNX27 in different brain regions of h*SNX27* Tg mice and found higher SNX27 expression in cerebral cortex and hippocampus, but not cerebellum, in h*SNX27* Tg mice than in WT littermates (Figures 1B,C). Furthermore, our immunohistochemical analysis indicated higher SNX27 expression in the cortical and hippocampal neurons of h*SNX27* Tg mice compared to that in WT mice (Figure 1D and Supplementary Figures 1D,E).

### Overexpression of Human SNX27 Ameliorated Anxiety-Like Behavior and Sociability

**FIGURE 2:**
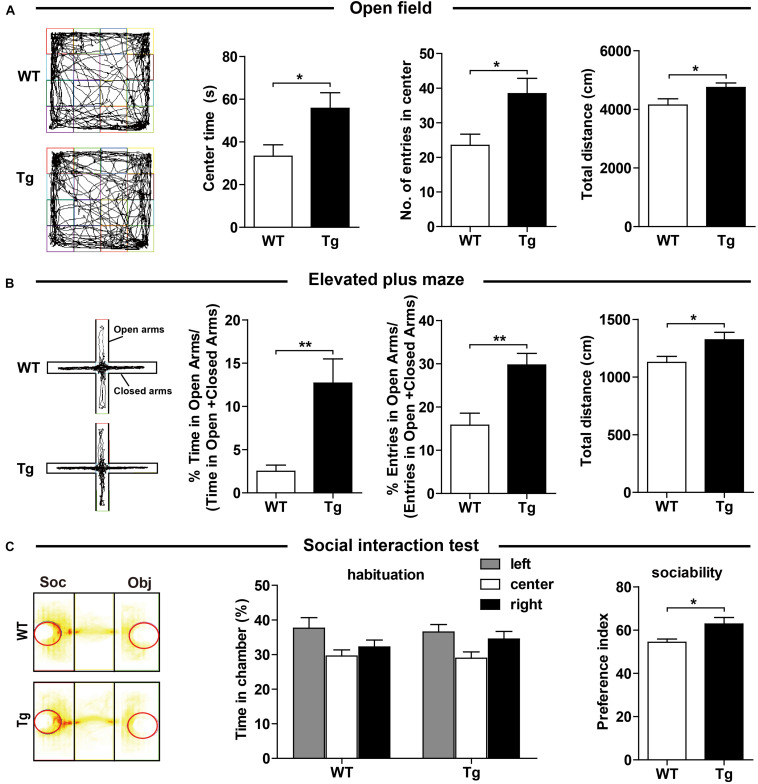
Effects of SNX27 overexpression on anxiety-like behavior and social interaction. **(A)** Time spent in the center, number of entries into the center, and distance traveled in open field test were quantified and analyzed. **(B)** In the elevated plus maze test, WT and Tg mice were assessed for percentage of time spent in the open arms, percentage of entries into the open arms, and total distance they traveled. **(C)** The durations of social interaction were measured in a 10-min social interaction test. Representative traces are shown. Data represent mean ± s.e.m. (WT, *n* = 14; Tg, *n* = 16). *P < 0.05, **P < 0.01 (two-tailed unpaired *t* test).

We carefully examined the h*SNX27* Tg mice and found that h*SNX27* Tg mice were grossly healthy, as the body weight gain and the ratio of genotypes were similar between h*SNX27* Tg mice and their WT littermates (Supplementary Figures 2A,B). To determine whether SNX27 overexpression affects mouse behavior, we performed a series of behavioral tests using 2–3-month-old mice. In the OFT, we found that h*SNX27* Tg mice spent more time in the central region of the open field compared to WT mice; furthermore, h*SNX27* Tg mice traveled a longer distance than WT mice did in the OFT (Figure 2A). In the EPM test, h*SNX27* Tg mice displayed markedly longer open-arm durations and more entry times compared to WT mice, and h*SNX27* Tg mice traveled longer distances than WT controls (Figure 2B). However, h*SNX27* Tg mice behaved similar to their WT littermates in the Light/Dark transition test (Supplementary Figure 3A). Interestingly, compared to WT mice, h*SNX27* Tg mice displayed enhanced sociability in the three-chamber test (Figure 2C). Taken together, these results indicate that SNX27 overexpression increases social interaction and reduces anxiety-like behavior in h*SNX27* Tg mice.

### Overexpression of Human SNX27 Enhanced Spatial Memory

**FIGURE 3:**
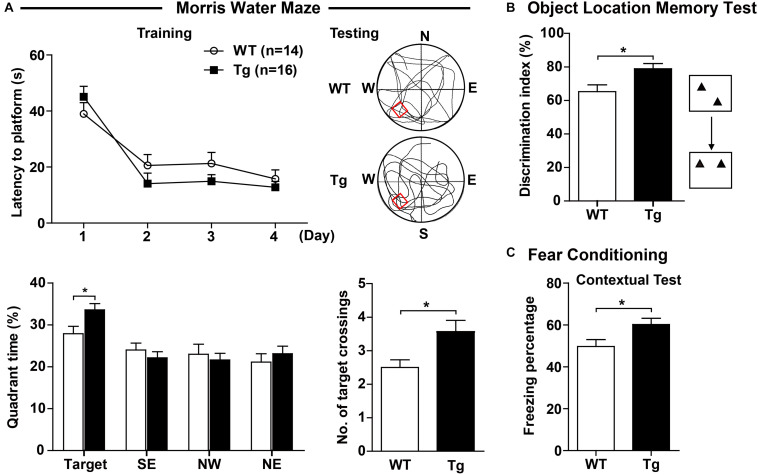
SNX27 overexpression enhanced learning and memory in h*SNX27* mice. **(A)** In the Morris water maze test, WT and Tg mice were evaluated for escape latency over a 4-day training. On the 5th day, percentage of time the mice spent in the target zone and the number of crossings over the platform region were quantified. **(B)** The object location memory test was conducted to assess the short-term (2 h) memory of WT and Tg mice. **(C)** In the fear conditioning test, freezing percentage was assessed to evaluate contextual memory 24 h after training. Representative traces are shown. Data represent mean ± s.e.m. (WT, *n* = 14; and Tg, *n* = 16). *P < 0.05 (two-tailed unpaired *t* test).

Sorting nexin 27 is highly expressed in hippocampal neurons, suggesting a potential role in learning and memory ability, we therefore evaluated learning and memory of h*SNX27* Tg mice. In the training phase of the MWM test, h*SNX27* Tg mice behaved similar to WT mice (Figure 3A). However, in the probe test of MWM, h*SNX27* Tg mice spent significantly more time in the target quadrant, underwent more platform crossings and required less time to reach the platform region than WT controls (Figure 3A and Supplementary Figure 3B), indicating that SNX27 overexpression enhances spatial memory in mice. However, there was no difference in total distance traveled between h*SNX27* Tg and WT mice (Supplementary Figure 3B). In the novel object recognition test, h*SNX27* Tg transgenic mice did not spend more time than WT mice in sniffing/exploring the novel object (Supplementary Figure 3C). However, we observed that h*SNX27* Tg mice yielded a higher discrimination index than WT controls in the novel object location test (Figure 3B). In addition, h*SNX27* Tg mice displayed significantly higher freezing percentages than WT mice in the fear conditioning contextual test (Figure 3C) but showed no difference from WT mice in the cued test (Supplementary Figure 3D). Together, these results indicate that SNX27 overexpression enhances learning and memory in mice.

### h*SNX27* Mice Displayed Enhanced Synaptic Function

**FIGURE 4:**
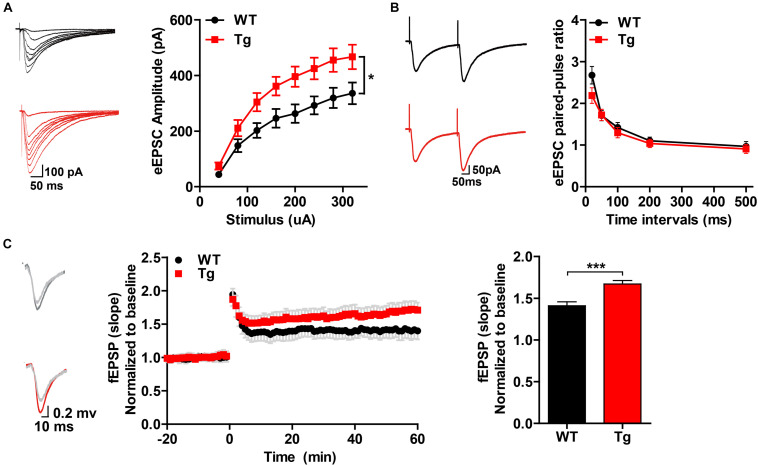
Overexpression of human SNX27 promoted synaptic plasticity in h*SNX27* mice. **(A)** Input–output response generated by stimulating the Schaffer-collateral pathway and recording in CA1 pyramidal neurons. (**A**, left) Representative traces of eEPSCs and quantitative analysis of eEPSC amplitude against stimulus intensity (**A**, right). eEPSCs were recorded at a holding potential of –70 mV. *n* = 6–8 cells per group. **(B)** Paired-pulse ratio analysis. (**B**, left) Representative traces of paired eEPSCs. (**B**, right) Summary of paired-pulse ratio against different time intervals. *n* = 6–8 cells per group. **(A,B)** Data represent mean ± s.e.m. *P < 0.05 (repeated measures two-way ANOVA). **(C)** LTP was induced in the CA3-CA1 synapses. (**C**, left) Representative traces of fEPSP responses before and after high frequency stimulation (HFS). (**C**, middle) Time course of LTP are shown for WT and Tg mice; LTP was induced by two trains of 100-Hz stimuli in the Schaffer-collateral pathway. (**C**, right) Quantitative analysis of fEPSP potentiation was determined at a mean of 50–60 min after high frequency stimulation. *n* = 5–7 slices per group. Data represent mean ± s.e.m. *P < 0.05, ***P < 0.001 (two-tailed unpaired *t* test).

**FIGURE 5:**
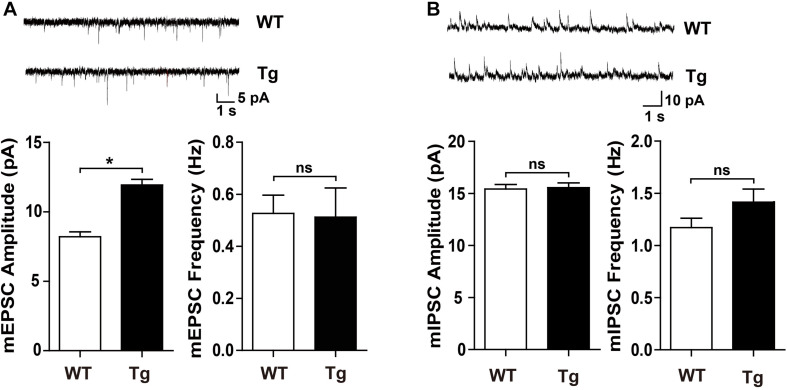
h*SNX27* transgenic mice displayed enhanced excitatory synaptic function. **(A)** (**A**, top) Representative traces of mEPSCs. mEPSCs were recorded at a holding potential of –70 mV. Scale bar = 1 s, 5 pA. (**A**, bottom) Quantitative analysis of the amplitude (left) and frequency (right) of mEPSCs. *n* = 15 cells per group. Data represent mean ± s.e.m. **(B)** (**B**, top) Representative traces of mIPSCs. mIPSCs were recorded at a holding potential of 0 mV. Scale bar = 1 s, 10 pA. (**B**, bottom) Quantitative analysis of the amplitude (left) and frequency (right) of mIPSCs. *n* = 20 cells per group. Data represent mean ± s.e.m. ns, not significant, *P < 0.05 (two-tailed unpaired *t* test).

To determine whether SNX27 overexpression affects synaptic function, we performed electrophysiological recordings using acute hippocampal slices from WT and h*SNX27* mice. In input-output response, a significant increase in the amplitude of the evoked excitatory postsynaptic current (eEPSC) was detected in h*SNX27* Tg mice relative to WT mice (Figure 4A). In addition, paired-pulse facilitation remains unchanged in h*SNX27* Tg mice compared to WT mice (Figure 4B). Consistent with input-output response, enhanced LTP at hippocampal CA1 region was observed in h*SNX27* mice compared to WT mice (Figure 4C), suggesting that SNX27 overexpression increases activity-dependent synaptic plasticity in hippocampus. Furthermore, h*SNX27* Tg mice showed enhanced mEPSC amplitude but unaffected mEPSC frequency compared to WT mice (Figure 5A), suggesting that SNX27 overexpression enhances the amount and/or the function of postsynaptic AMPA and NMDA receptors. However, both amplitude and frequency of mIPSC were similar between h*SNX27* Tg mice and WT littermates (Figure 5B). Moreover, excitation/inhibition balance (E/I ratio) was not affected by SNX27 overexpression in hSNX27 transgenic mice (Supplementary Figure 4). Together, these results demonstrate that SNX27 overexpression enhances excitatory synaptic transmission and synaptic plasticity in h*SNX27* Tg mice.

### Overexpression of Human SNX27 Affected Synapse Formation

**FIGURE 6:**
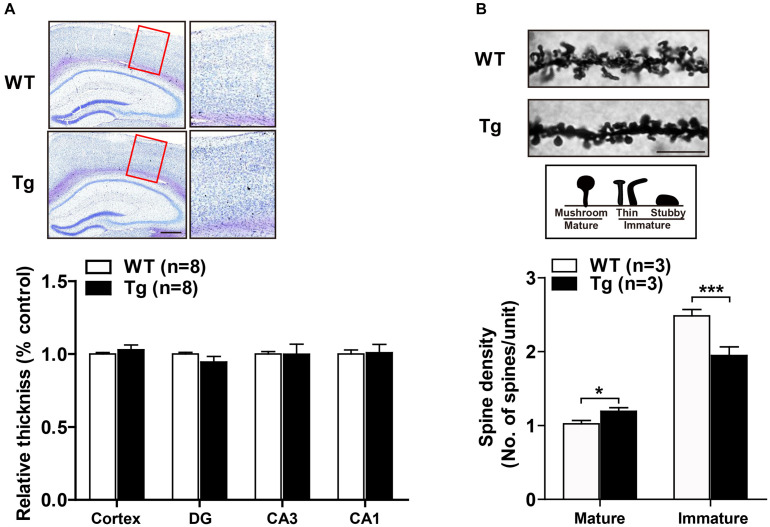
Effects of SNX27 overexpression on synaptogenesis. **(A)** (**A**, top) Representative images of Nissl staining of WT and Tg brain slices. The zoom in regions were marked by a red box. Scale bar = 400 μm. (**A**, bottom) Cortical thickness. *n* = 8. **(B)** (**B**, top) Representative images and illustrations of Golgi staining of hippocampal neurons of 2-month-old WT and Tg mice. Scale bar = 10 μm. (**B**, bottom) Spine densities of mature (mushroom) and immature (stubby and thin) neurons were quantified. Data represent mean ± s.e.m. (*n* = 21–30 neurons from three mice per group). *P < 0.05, ***P < 0.001 (two-tailed unpaired *t* test).

Enhanced synaptic function is usually accompanied by dynamic alterations in synaptic density or structure, such as the formation of new synapses or the consolidation of existing synapses (Batool et al., 2019). We first performed Nissl staining and found that SNX27 overexpression did not affect the gross morphology of cortex and hippocampus of h*SNX27* Tg mice (Figure 6A). To determine whether SNX27 overexpression affects synapse formation, we consequently performed Golgi staining and found an increased density of mature synapses and a decreased number of immature synapses in hippocampal neurons of 2-month-old hS*NX27* Tg mice compared with that in WT littermates (Figure 6B), suggesting that SNX27 plays a key role in regulating synaptic structure and function without affecting gross brain morphology.

### Overexpression of Human SNX27 Upregulated the Expression of Synaptic Proteins

**FIGURE 7:**
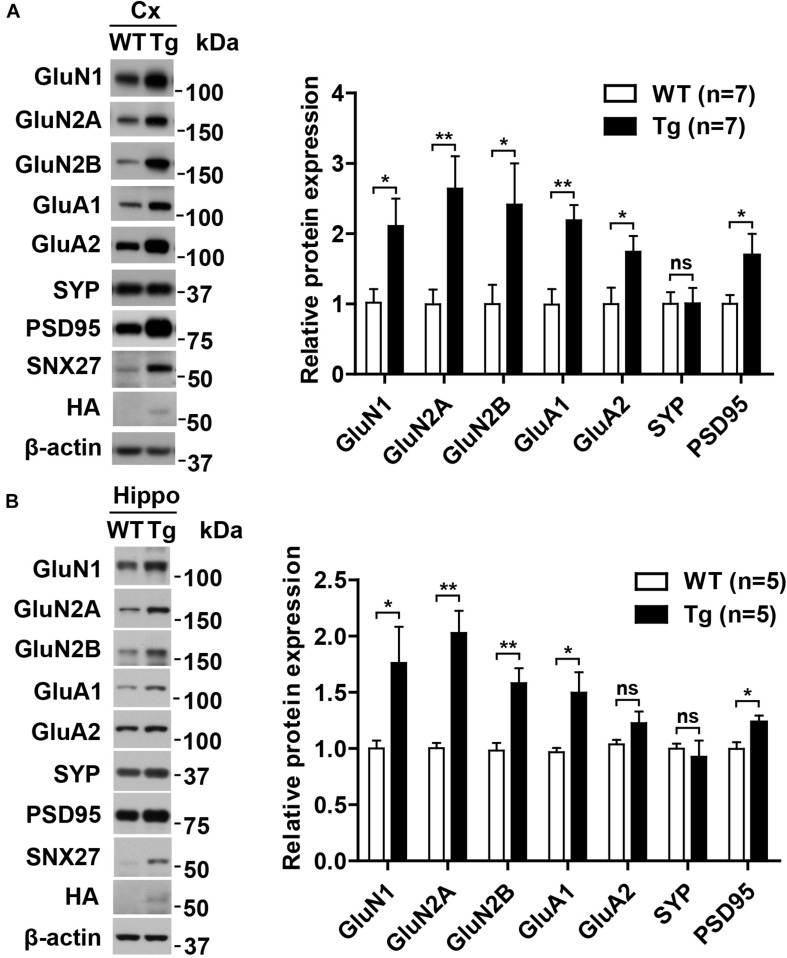
Upregulation of NMDA and AMPA receptors in h*SNX27* transgenic mice. **(A,B)** Total lysates from the cortex **(A)** and hippocampus **(B)** of 2-month-old WT and Tg mice were analyzed by western blot analysis. Protein levels were normalized to β-actin compared with WT. Data represent mean ± s.e.m. (*n* = 3 per group). ns, not significant, *P < 0.05, **P < 0.01 (two-tailed unpaired *t* test).

To explore the underlying mechanism of enhanced synaptic function in h*SNX27* Tg mice, we analyzed the expression of synaptic proteins, including glutamate receptors and postsynaptic scaffold proteins, and found an upregulated expression of glutamate receptors and the scaffold protein PSD95 in both cortex and hippocampus of h*SNX27* Tg mice compared with WT controls (Figures 7A,B). However, the mRNA levels of glutamate receptors did not differ between the two groups (Supplementary Figure 5), suggesting that SNX27 overexpression modulates the expression of these receptors at the post-transcriptional level. In addition, SNX27 overexpression failed to affect the expression levels of inhibitory synaptic proteins, such as GABA_*A*_Rβ3 and GAD-65 (Supplementary Figure 6). Taken together, these results indicate that SNX27 overexpression increases excitatory synaptic function by upregulating the expression of glutamate receptors and PSD-associated proteins in mice.

### h*SNX27* Transgenic Mice Did Not Display Seizure-Like Electroencephalogram (EEG)

**FIGURE 8:**
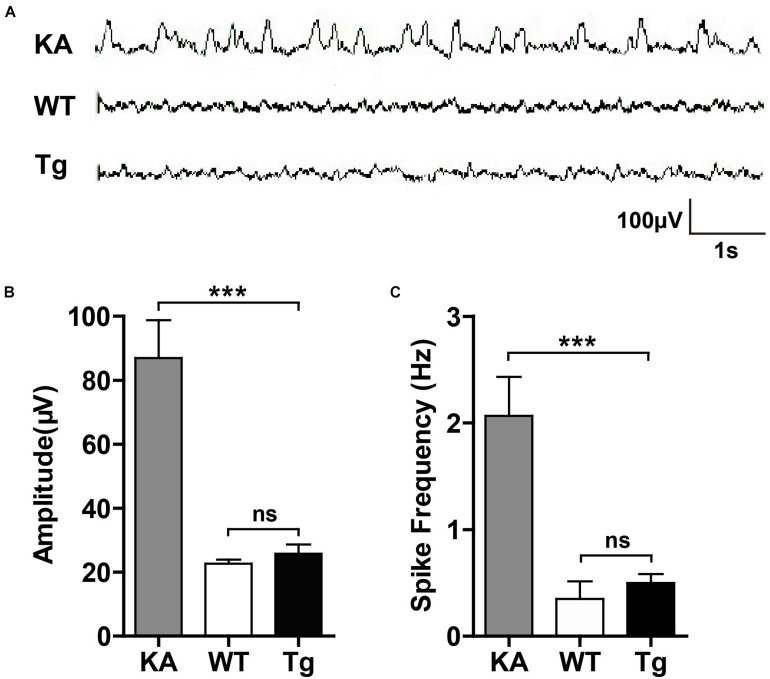
SNX27 overexpression did not result in seizure-like EEG. **(A)** Representative EEG images. 2-month-old WT and Tg mice were recorded for EEG for 1 h. C57BL/6 mice were injected with KA as positive control and subjected to EEG recording for 2 h after injection. **(B,C)** The amplitude **(B)** and spike frequency **(C)** of EEG were quantified for comparison. *n* = 3 mice. Data represent mean ± s.e.m. (*n* = 3 per group). ns, not significant, ***P < 0.001 (one-way ANOVA with Turkey *post hoc* analysis).

Given that hyperactivation of glutamate receptors may lead to excessive firing of neurons, we examined whether SNX27 overexpression results in epilepsy-like EEG (Figure 8A). In contrast to an induced seizure model (KA-injected mice), neither h*SNX27* Tg nor WT mice displayed seizure-like episodes and EEG abnormalities (Figures 8B,C). Together, these results suggest that SNX27 overexpression does not induce seizure-like behavior.

## Discussion

Impaired synaptic function contributes to various neurological diseases, including autism spectrum disorders (ASD), DS, epilepsy and AD (Terry et al., 1991; Kril et al., 2002; Fernandez and Garner, 2007; Baudouin et al., 2012; Keezer et al., 2016; DeTure and Dickson, 2019; Guo et al., 2020). In our previous work, we found that SNX27 haploinsufficiency disrupts glutamate receptor recycling, leading to synaptic and cognitive deficits (Wang et al., 2013). In the current study, we further determined that overexpression of SNX27 enhances synaptic and cognitive function in *hSNX27* Tg mice. These findings demonstrate that SNX27 plays a key role in synaptic plasticity and cognitive function through regulating intracellular trafficking of inotropic glutamate receptors.

Previous studies have shown that the SNX27-retromer-WASH complex is involved in endosomal sorting and trafficking of protein cargoes. SNX27 serves as an adaptor protein to link multiple cargo proteins with the endosomal tubules through direct interactions. In addition, SNX27 interacts with VPS26 (a subunit of the retromer complex) through its PDZ domain to prevent lysosomal delivery and degradation of its cargoes (Seaman, 2004; Steinberg et al., 2013; Lee et al., 2016). In addition to glutamate receptors, future work will illustrate whether other cargo proteins of SNX27 are involved in the regulation of synaptic and cognitive function.

Synaptic function can be also regulated by the extracellular matrix and glial cells, which are referred to as the “tetrapartite synapse” (Chelini et al., 2018). In addition to neurons, SNX27 also plays important roles in normal function of glial cells. SNX27 is expressed in oligodendrocytes and mediates the intracellular delivery of GPR17 to the plasma membrane, and participates in the development of oligodendrocytes and myelin sheath (Meraviglia et al., 2016). In our study, we also confirmed the expression of SNX27 in astrocytes. Given that astrocytes act as the supporting cells which provides lactate/pyruvate to neurons, and the roles of astrocytes in regulating synapse maturation and synaptic plasticity (Bosworth and Allen, 2017; Blanco-Suarez et al., 2018; Hillen et al., 2018; Scofield, 2018), SNX27 may regulates synaptic plasticity through modulating astrocytic function, and the cell-type-specific functions of SNX27 require careful investigation in the future studies.

Human-*SNX27* Tg mice exhibited altered social and anxiety-like behavior. It has been reported that abnormal NMDAR activity contributes to impaired social interaction and anxiety-like behavior in mice (Planaguma et al., 2015; Zhang et al., 2016; Peyrovian et al., 2019). In the future study, the mechanisms by which SNX27 influences anxiety-like behavior need further investigation.

In summary, our results indicate that SNX27 promotes glutamate receptor recycling and enhances synaptic and cognitive function without inducing seizure-like behavior and EEG in h*SNX27* transgenic mice. Our results suggest SNX27 as a potential therapeutic target for treating intellectual disability and other neurodegenerative diseases.

## Data Availability Statement

The original contributions presented in the study are included in the article/Supplementary Material, further inquiries can be directed to the corresponding author.

## Ethics Statement

The animal study was reviewed and approved by the Institutional Animal Care and Use Committee of Xiamen University.

## Author Contributions

XW conceptualized the study and designed the experiments. YH performed the behavioral tests and biochemical assays. DZ, TG, SZ, and YZe performed the morphological analysis. YG and YC performed the electrophysiological recording. QZ, HG, LZ, and BZ performed the molecular biology experiments. Y-wZ and HS supervised DZ and YG, respectively. YH and XW wrote the manuscript. DZ, HL, XZ, YZh and HX discussed and edited the manuscript. XW supervised the project. All authors contributed to the article and approved the submitted version.

## Conflict of Interest

The authors declare that the research was conducted in the absence of any commercial or financial relationships that could be construed as a potential conflict of interest.

## Abbreviations

AD: Alzheimer’s disease
AMPA: α-Amino-3-hydroxy-5-methyl-4-isoxazolepropionic acid
APP: amyloid precursor protein
Camk2a: Calcium/Calmodulin Dependent Protein Kinase II Alpha
DS: Down’s syndrome
EEG: electroencephalogram
GABA_*A*_R β 3: γ-aminobutyric acid type A receptor β 3 subunit
GAD-65: glutamic acid decarboxylase 65-kilodalton isoform
KA: Kainic acid
LTP: long-term potentiation
mEPSC: miniature excitatory postsynaptic current
mIPSC: miniature inhibitory postsynaptic current
NMDA: *N*-methyl-D-aspartate
PFA: Paraformaldehyde
PVDF: polyvinylidene difluoride
PX domain: the Phox homology domain
RA domain: the Ras-association domain
SNX27: Sorting nexin 27
Tg: Transgenic
WT: Wild-type.
