# Supplementary material for: Overexpression of Human SNX27 Enhances Learning and Memory Through Modulating Synaptic Plasticity in Mice
Source: Front Cell Dev Biol. 2020 Nov 27;8:595357. doi: 10.3389/fcell.2020.595357 (PMC7729021; doi:10.3389/fcell.2020.595357)
Supplement: Supplementary file 1 [file Table_1.docx]

**Supplementary Figures**


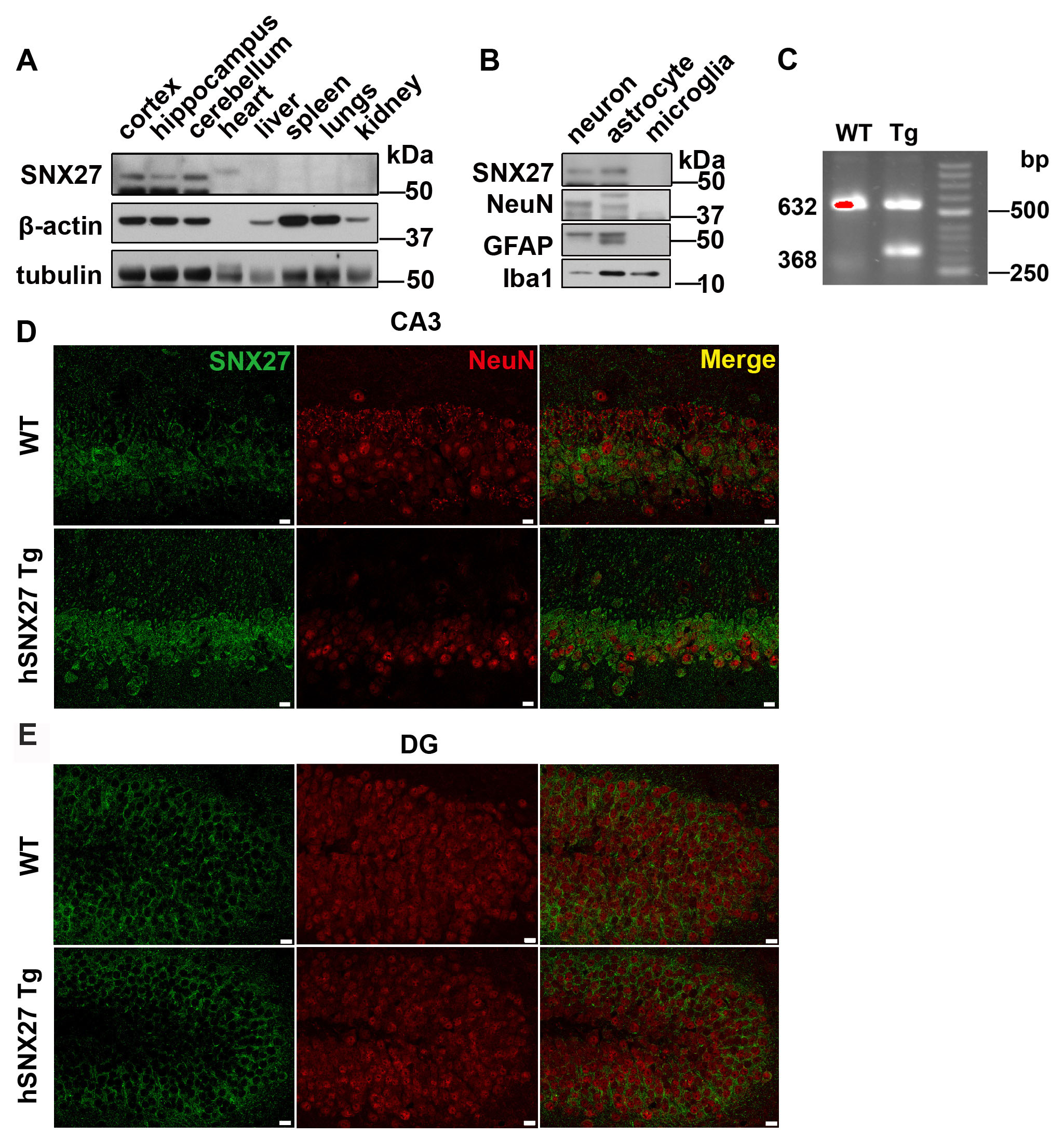


**Supplementary** **Figure S1 | Expression pattern of SNX27 in WT and h*SNX27* transgenic mice**. **(A, B)** Expression levels of endogenous SNX27 in the brain and other tissues of adult C57BL/6 mice (**A**) and in different cell types **(B)** were determined by western blot. **(C)** Genotyping of WT and h*SNX27* transgenic mice. Amplified PCR products spanning the targeted region were subjected to agarose gel analysis to resolve WT and Tg fragments. Tg, h*SNX27* transgenic mice. **(D, E)** Expression pattern of human SNX27 in CA3 **(D)** and DG **(E)** regions of h*SNX27* transgenic mice were determined by immunofluorescence staining. Slices were immunostained with SNX27 (in green) and NeuN (in red) antibodies and observed by confocal microscopy. WT mice were used as negative controls. Scale bar = 10 μm.

**Supplementary** **Figure S2 |** SNX27 transgenic mice were grossly healthy. **(A)** Body weight gain of WT and Tg mice over 60 days (*n* = 15). **(B)** The offspring of h*SNX27* transgenic mice were scored by genotype and sex at postnatal day 45.

**Supplementary** **Figure S3 |** Behavioral analysis of h*SNX27* transgenic mice. **(A)** Anxiety-like behavior in the dark/light transition test was measured as the percentage of time spent in the light chamber. **(B)** In the Morris water maze test, mice were assessed for latency to enter the platform area and total distance travelled on the 5th day. **(C)** In the novel object recognition test, WT and h*SNX27* transgenic mice were scored for exploration time under new object replacement at 24 h post training. **(D)** In the fear conditioning test, the percentage of freezing time was measured in the cued test. Data represent mean ± s.e.m. (WT, *n* = 14; Tg, *n* = 16). ns, not significant, **P* < 0.05, ***P* < 0.01 (two-tailed unpaired t test).

**
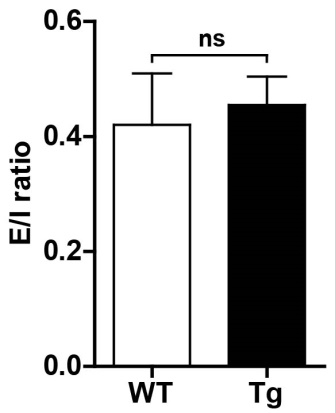
**

**Supplementary** **Figure S4** Excitation/inhibition ratio (E/I ratio) was unaffected in h*SNX27* transgenic mice. *n* = 15 cells from 3 Tg mice, *n* = 20 cells from 3 WT mice. Data shown as mean ± s.e.m. ns, not significant (two-tailed unpaired t test).


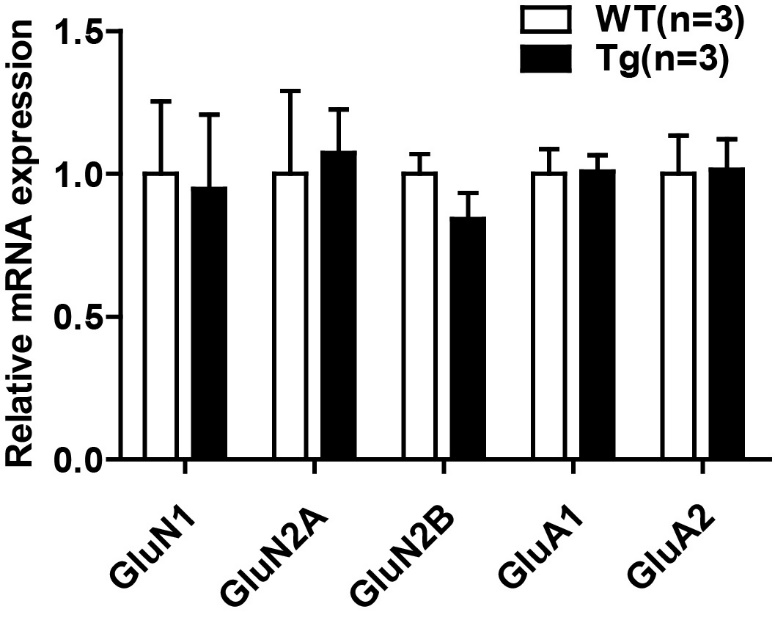


**Supplementary** **Figure S5 |** Relative mRNA expression of glutamate receptors in the cortex of h*SNX27* transgenic mice were determined by quantitative real-time PCR. Values were normalized to *Actb*. WT values were set as one arbitrary unit (*n* = 3).

**
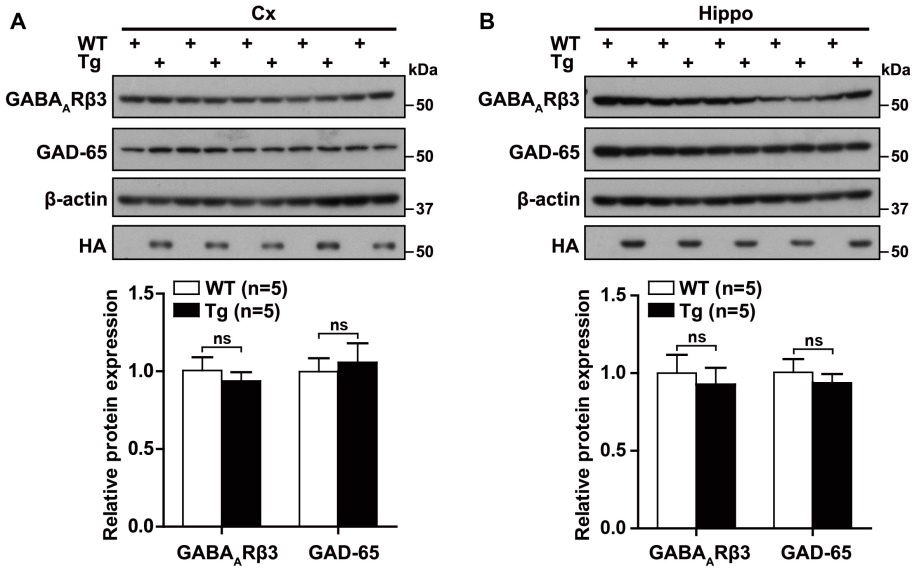
**

**Supplementary** **Figure S6 |** Expression levels of inhibitory synaptic proteins were unaffected in h*SNX27* transgenic mice. **(A, B)** Total lysates of the cortex **(A)** and hippocampus **(B)** from 2-month-old WT and Tg mice were analyzed by western blot analysis. Protein levels were normalized to β-actin compared with WT. Data represent mean ± s.e.m. (*n* = 5 per group). ns, not significant (two-tailed unpaired t test).
