# Supplementary figures and images for: Overexpression of Human SNX27 Enhances Learning and Memory Through Modulating Synaptic Plasticity in Mice
Source: Front Cell Dev Biol. 2020 Nov 27;8:595357. doi: 10.3389/fcell.2020.595357 (PMC7729021; doi:10.3389/fcell.2020.595357)

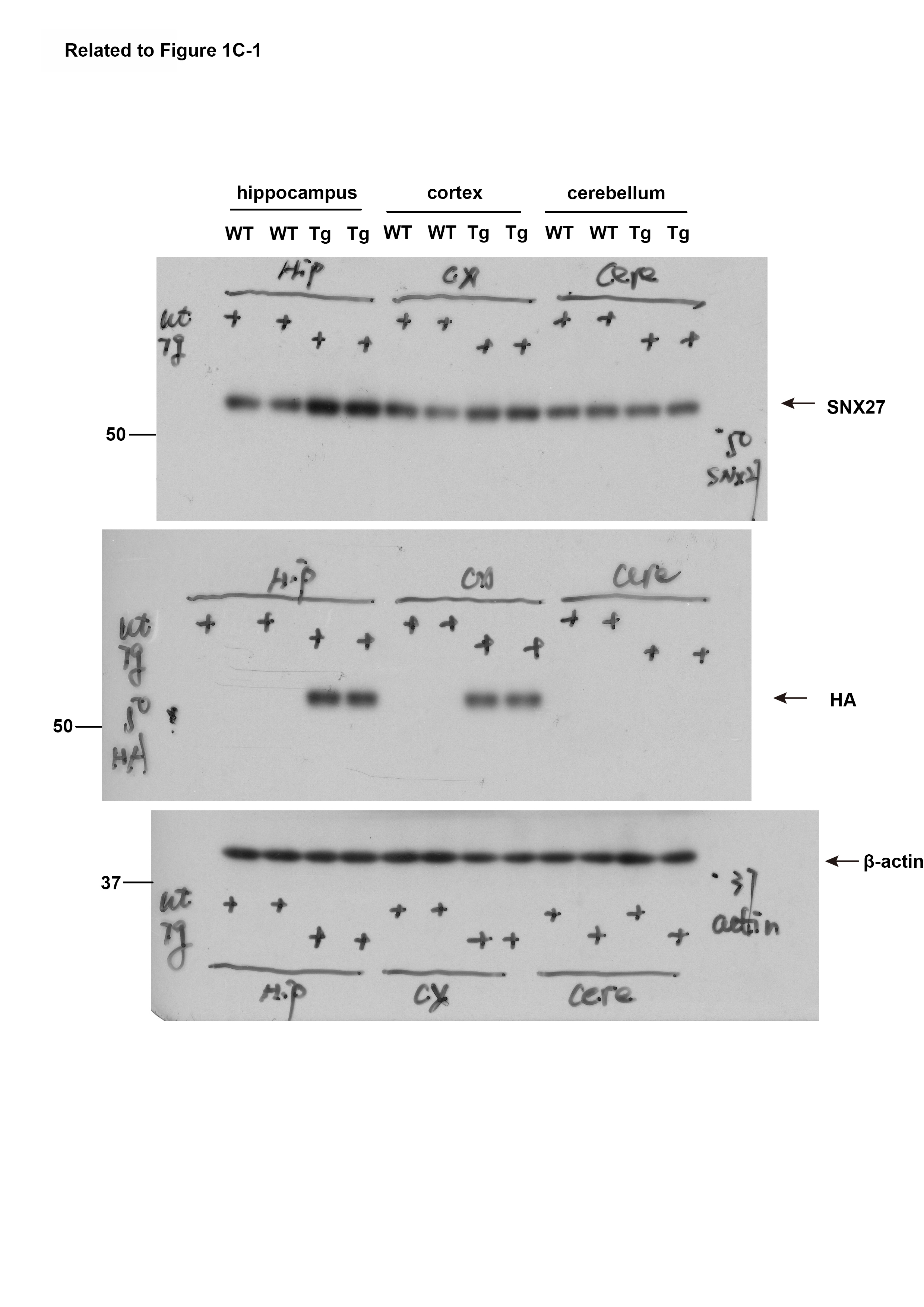

Supplement: Supplementary file 2 [file Data_Sheet_1.ZIP › raw data/Figure 1C-1.jpg]

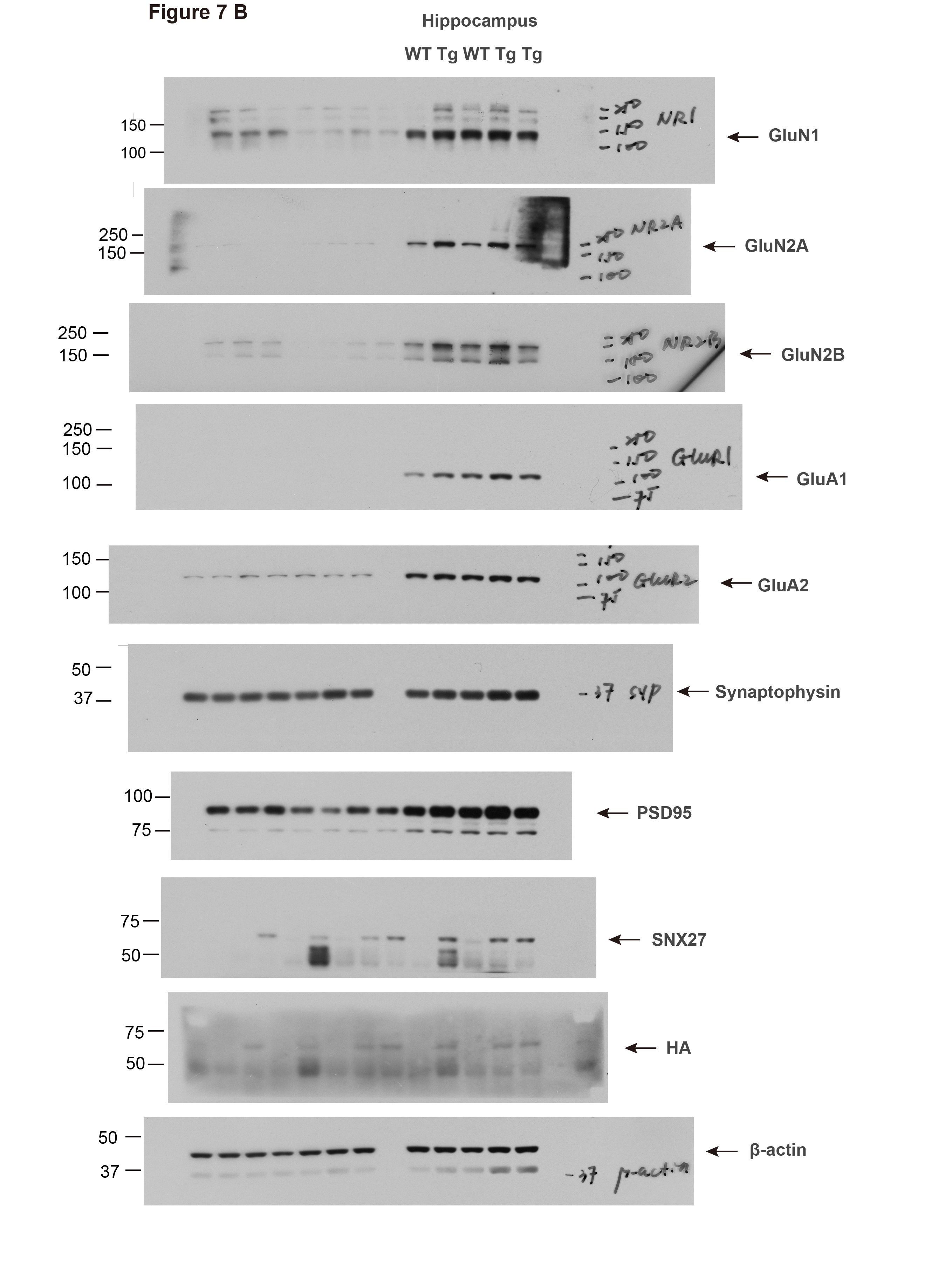

Supplement: Supplementary file 2 [file Data_Sheet_1.ZIP › raw data/Figure 7B.jpg]

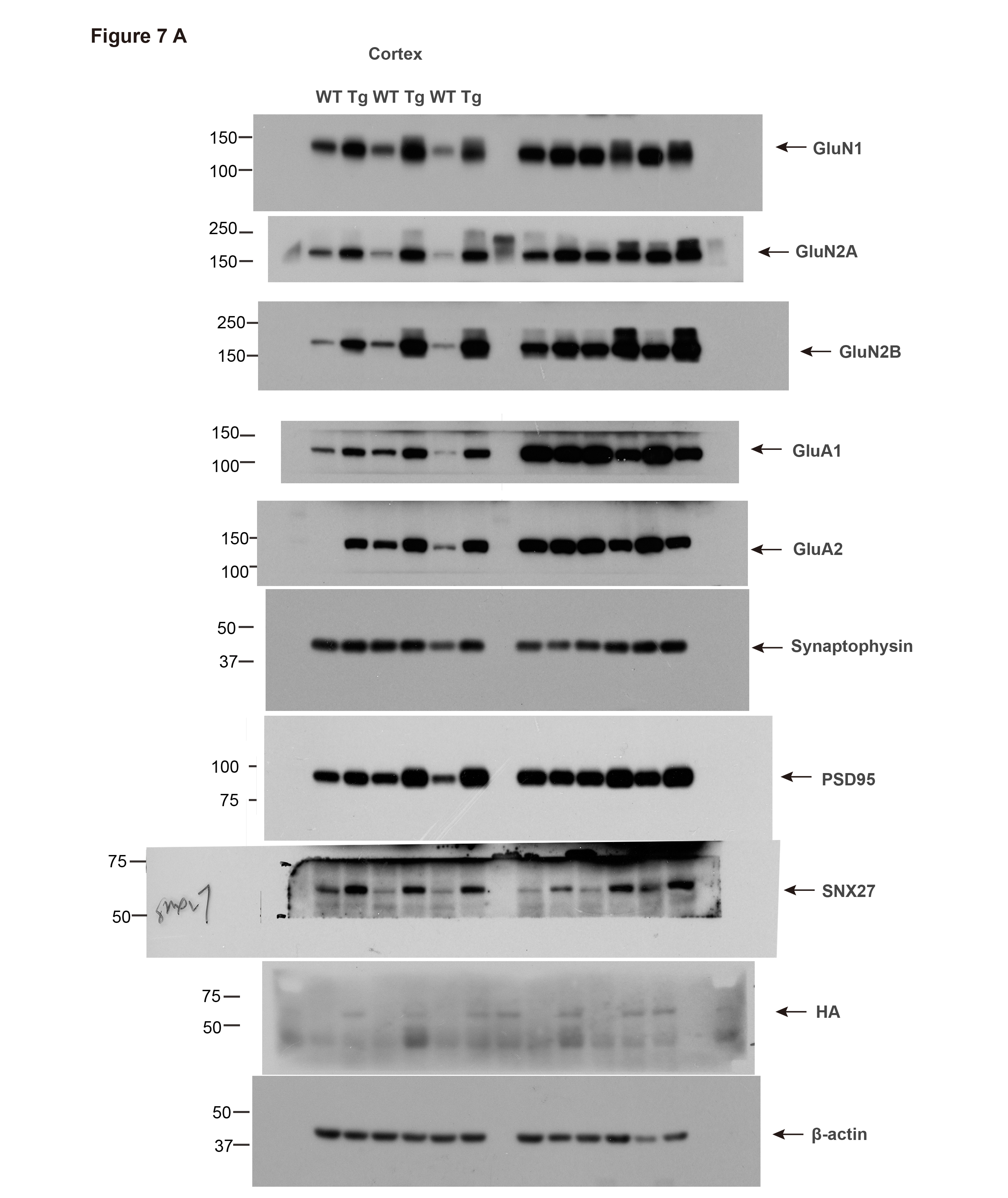

Supplement: Supplementary file 2 [file Data_Sheet_1.ZIP › raw data/Figure 7A.jpg]

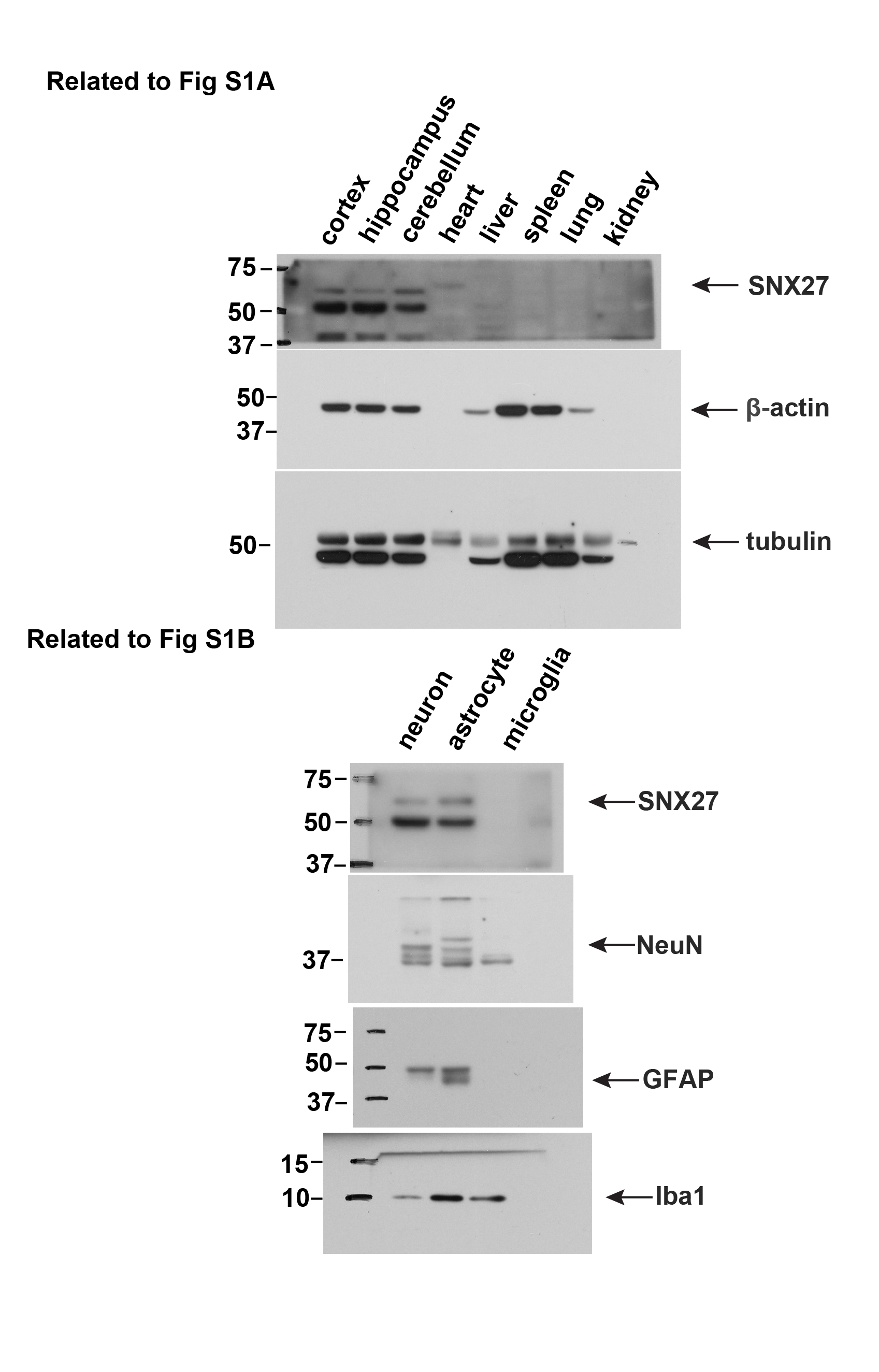

Supplement: Supplementary file 2 [file Data_Sheet_1.ZIP › raw data/Figure S1A-S1B.jpg]

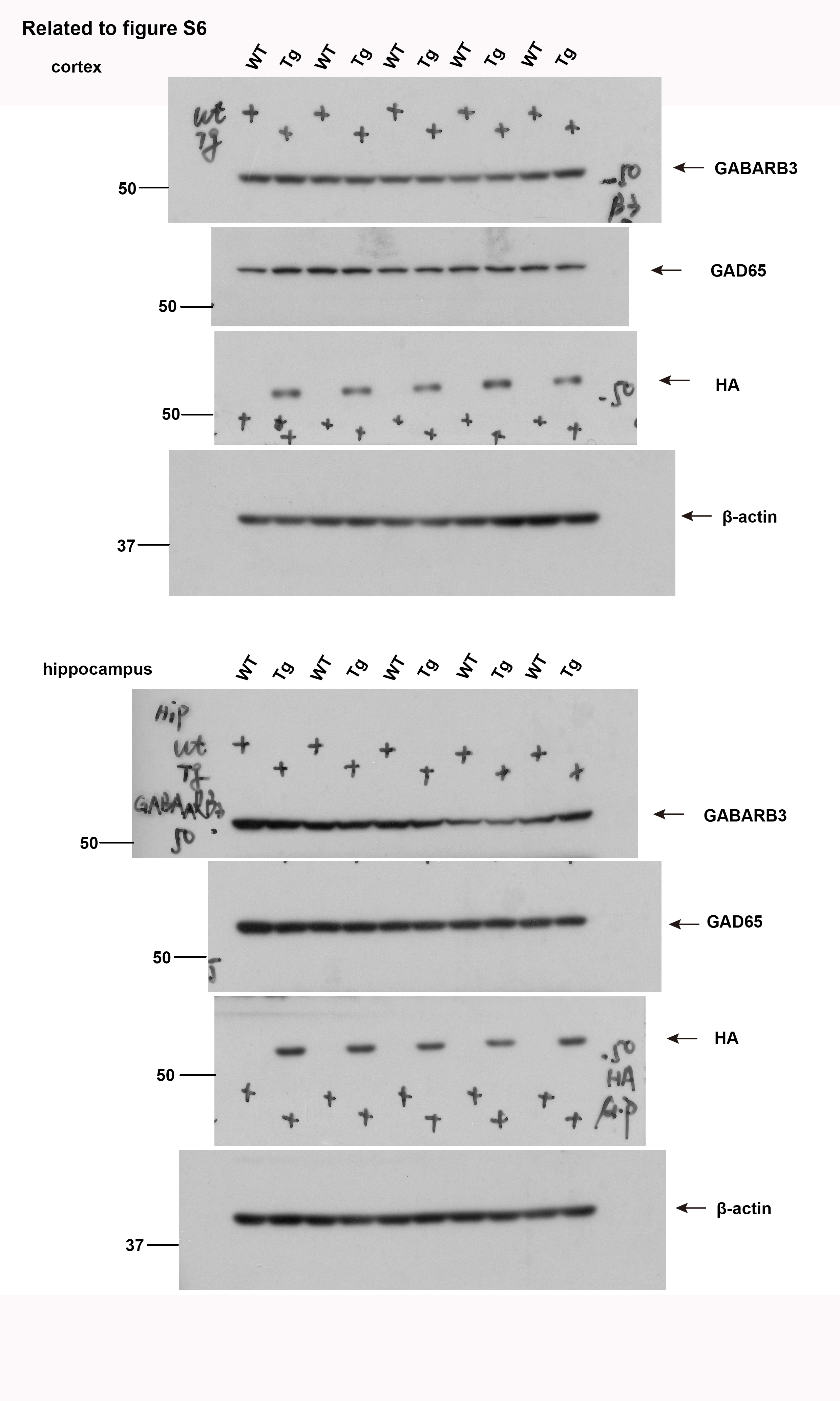

Supplement: Supplementary file 2 [file Data_Sheet_1.ZIP › raw data/Figure S6.jpg]

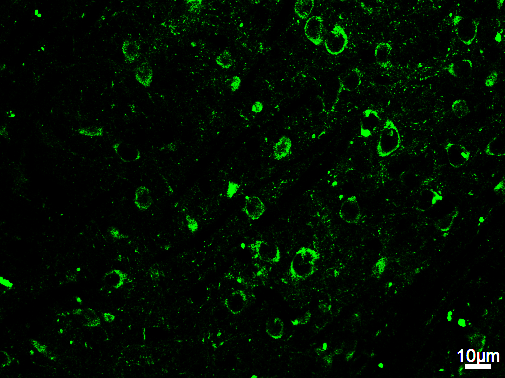

Supplement: Supplementary file 2 [file Data_Sheet_1.ZIP › raw data/wt/wt-cortex-60x-SNX27.tif]

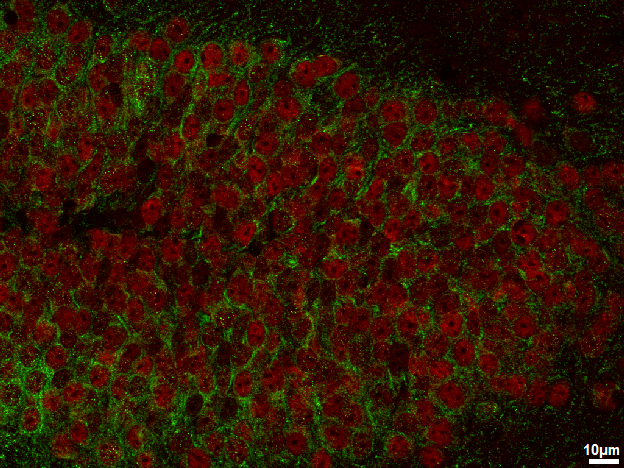

Supplement: Supplementary file 2 [file Data_Sheet_1.ZIP › raw data/wt/wt-DG-60x-merge.tif]

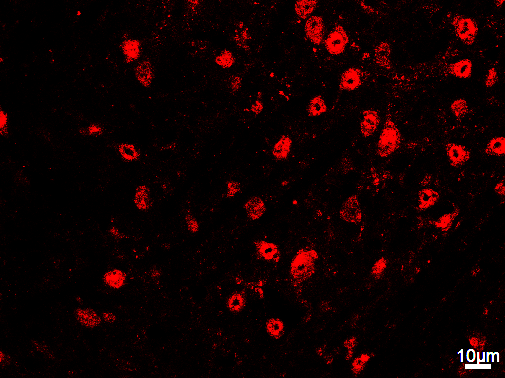

Supplement: Supplementary file 2 [file Data_Sheet_1.ZIP › raw data/wt/wt-cortex-60x-NeuN.tif]

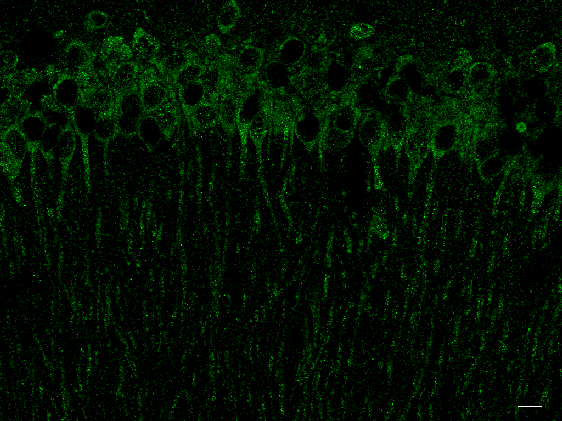

Supplement: Supplementary file 2 [file Data_Sheet_1.ZIP › raw data/wt/wt-CA1-3-60x-SNX27.tif]

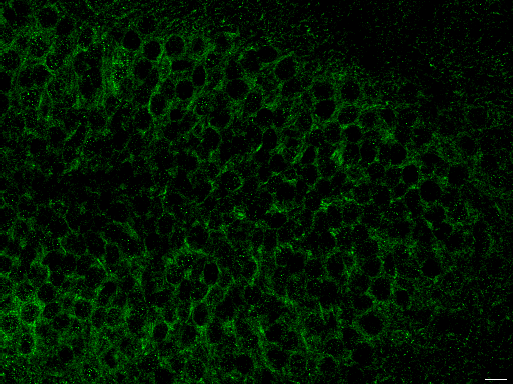

Supplement: Supplementary file 2 [file Data_Sheet_1.ZIP › raw data/wt/wt-DG-60-SNX27.tif]

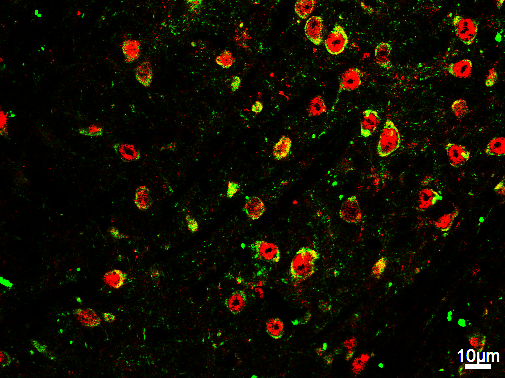

Supplement: Supplementary file 2 [file Data_Sheet_1.ZIP › raw data/wt/wt-cortex-60x-merge.tif]

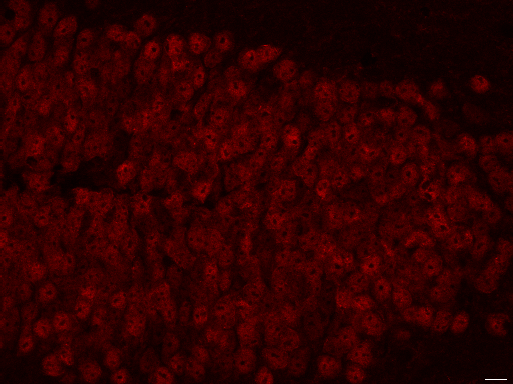

Supplement: Supplementary file 2 [file Data_Sheet_1.ZIP › raw data/wt/wt-DG-60x-NeuN.tif]

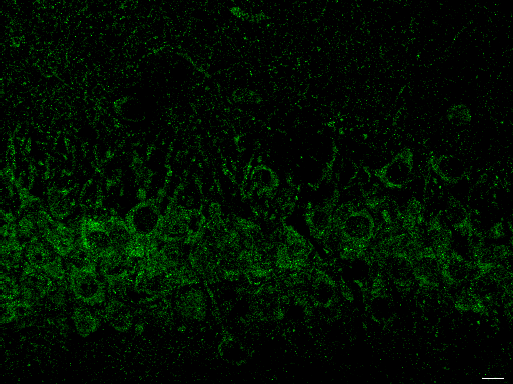

Supplement: Supplementary file 2 [file Data_Sheet_1.ZIP › raw data/wt/wt-CA3-1-60-SNX27.tif]

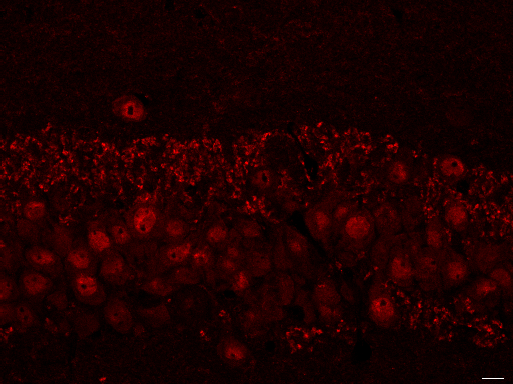

Supplement: Supplementary file 2 [file Data_Sheet_1.ZIP › raw data/wt/wt-CA3-1-60-NeuN.tif]

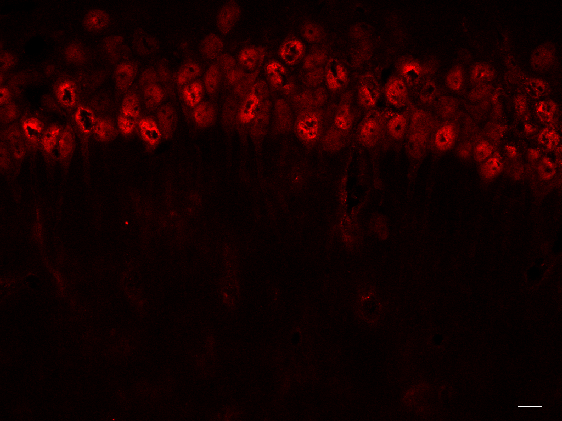

Supplement: Supplementary file 2 [file Data_Sheet_1.ZIP › raw data/wt/wt-CA1-3-60x-NeuN.tif]

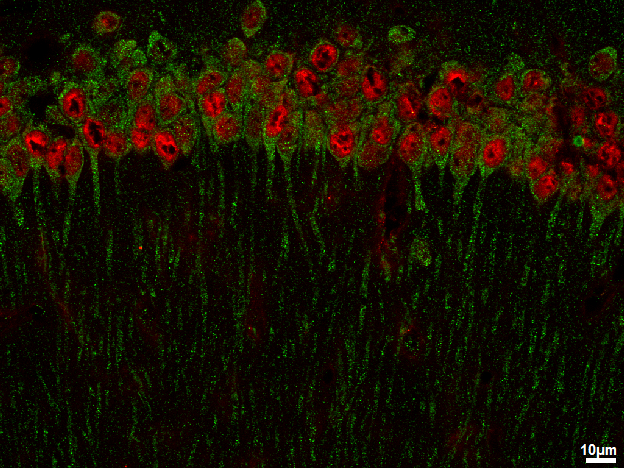

Supplement: Supplementary file 2 [file Data_Sheet_1.ZIP › raw data/wt/wt-CA1-3-60x-merge.tif]

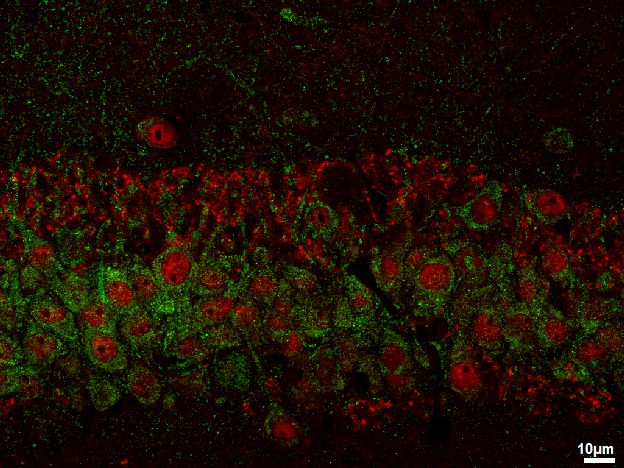

Supplement: Supplementary file 2 [file Data_Sheet_1.ZIP › raw data/wt/wt-CA3-1-60x-merge.tif]

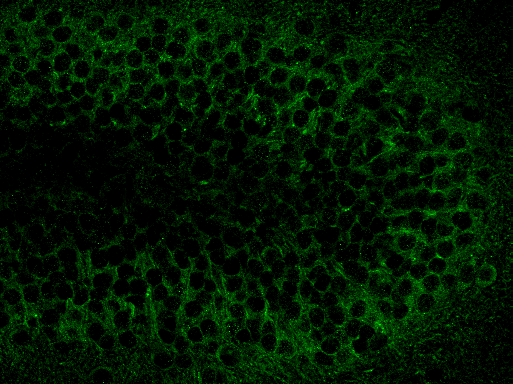

Supplement: Supplementary file 2 [file Data_Sheet_1.ZIP › raw data/tg/Tg-DG-60x-SNX27.tif]

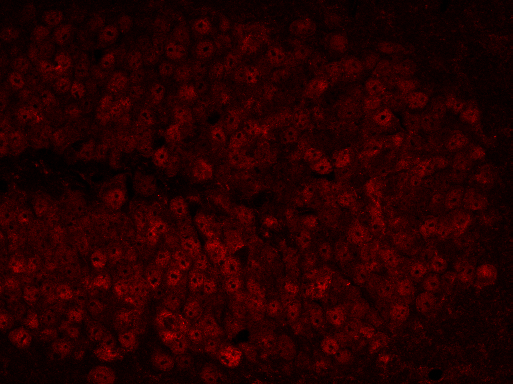

Supplement: Supplementary file 2 [file Data_Sheet_1.ZIP › raw data/tg/Tg-DG-60x-NeuN.tif]

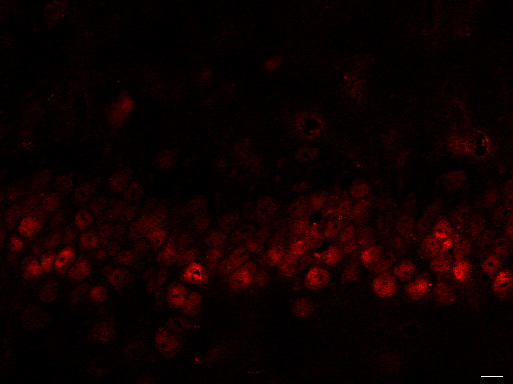

Supplement: Supplementary file 2 [file Data_Sheet_1.ZIP › raw data/tg/Tg-CA3-60-NeuN.tif]

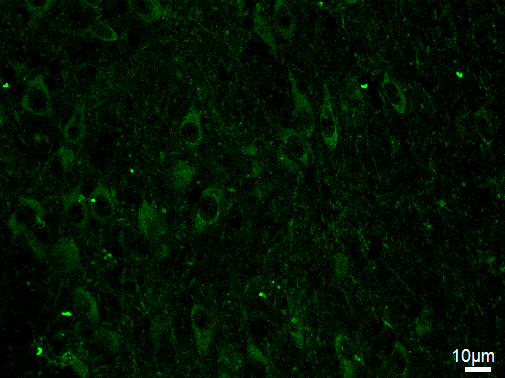

Supplement: Supplementary file 2 [file Data_Sheet_1.ZIP › raw data/tg/Tg-cortex-60x-SNX27.tif]

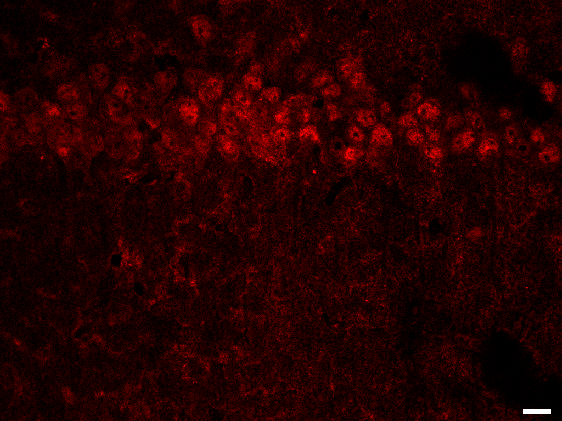

Supplement: Supplementary file 2 [file Data_Sheet_1.ZIP › raw data/tg/Tg-CA1-NeuN-60x.tif]

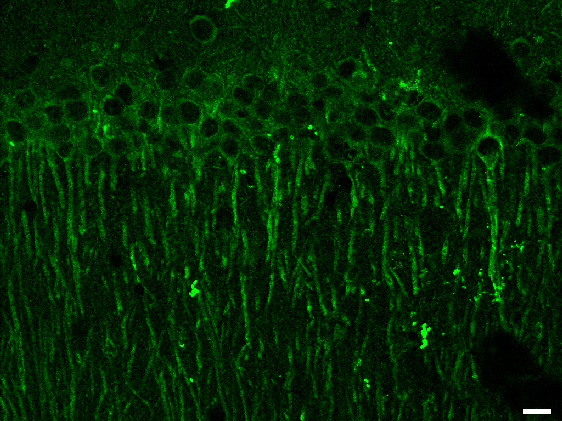

Supplement: Supplementary file 2 [file Data_Sheet_1.ZIP › raw data/tg/Tg-CA1-SNX27-60x.tif]

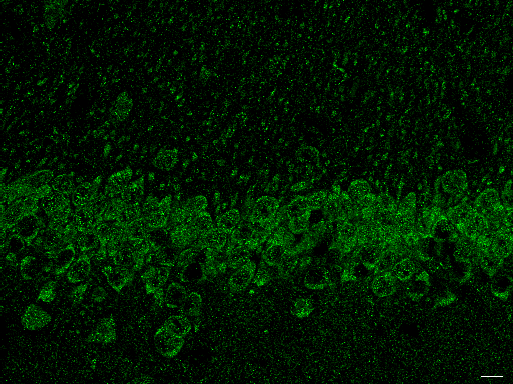

Supplement: Supplementary file 2 [file Data_Sheet_1.ZIP › raw data/tg/Tg-CA3-60X-SNX27.tif]

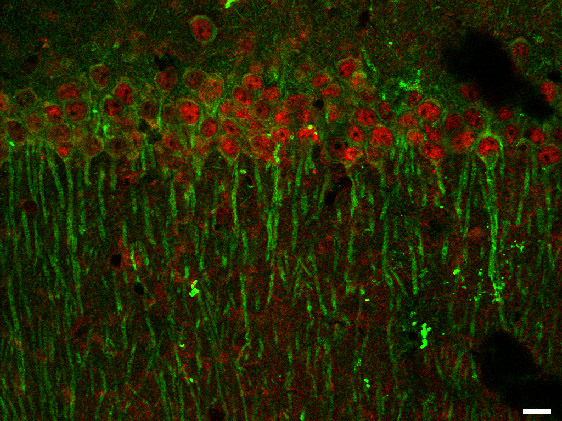

Supplement: Supplementary file 2 [file Data_Sheet_1.ZIP › raw data/tg/Tg-CA1-merge-60x.tif]

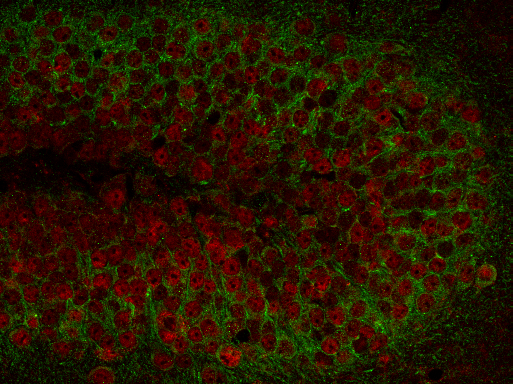

Supplement: Supplementary file 2 [file Data_Sheet_1.ZIP › raw data/tg/Tg-DG-60x-merge.tif]

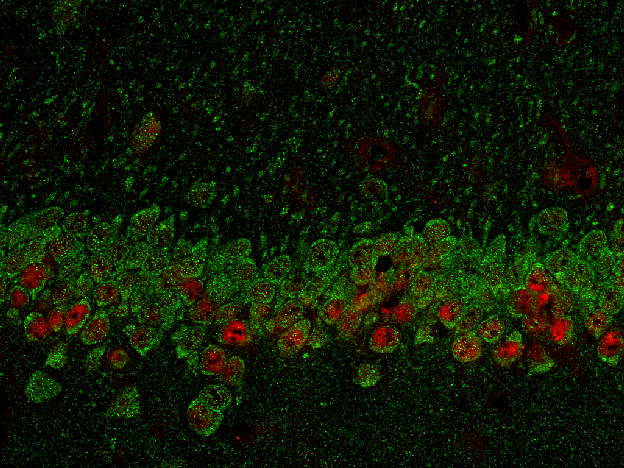

Supplement: Supplementary file 2 [file Data_Sheet_1.ZIP › raw data/tg/Tg-CA3-60X-merge.tif]

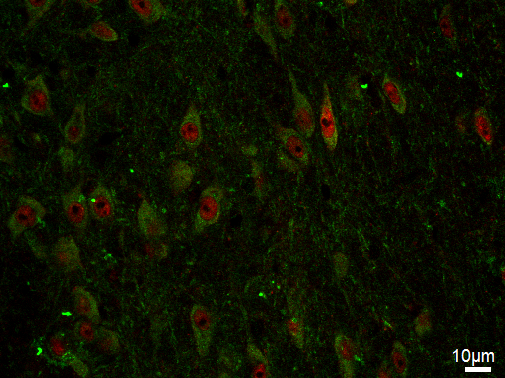

Supplement: Supplementary file 2 [file Data_Sheet_1.ZIP › raw data/tg/Tg-cortex-60x-merge.tif]

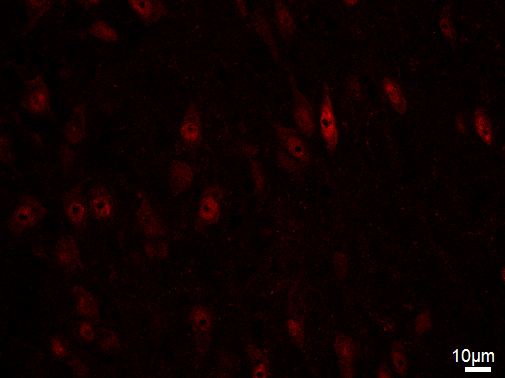

Supplement: Supplementary file 2 [file Data_Sheet_1.ZIP › raw data/tg/Tg-cortex-60x-NeuN.tif]

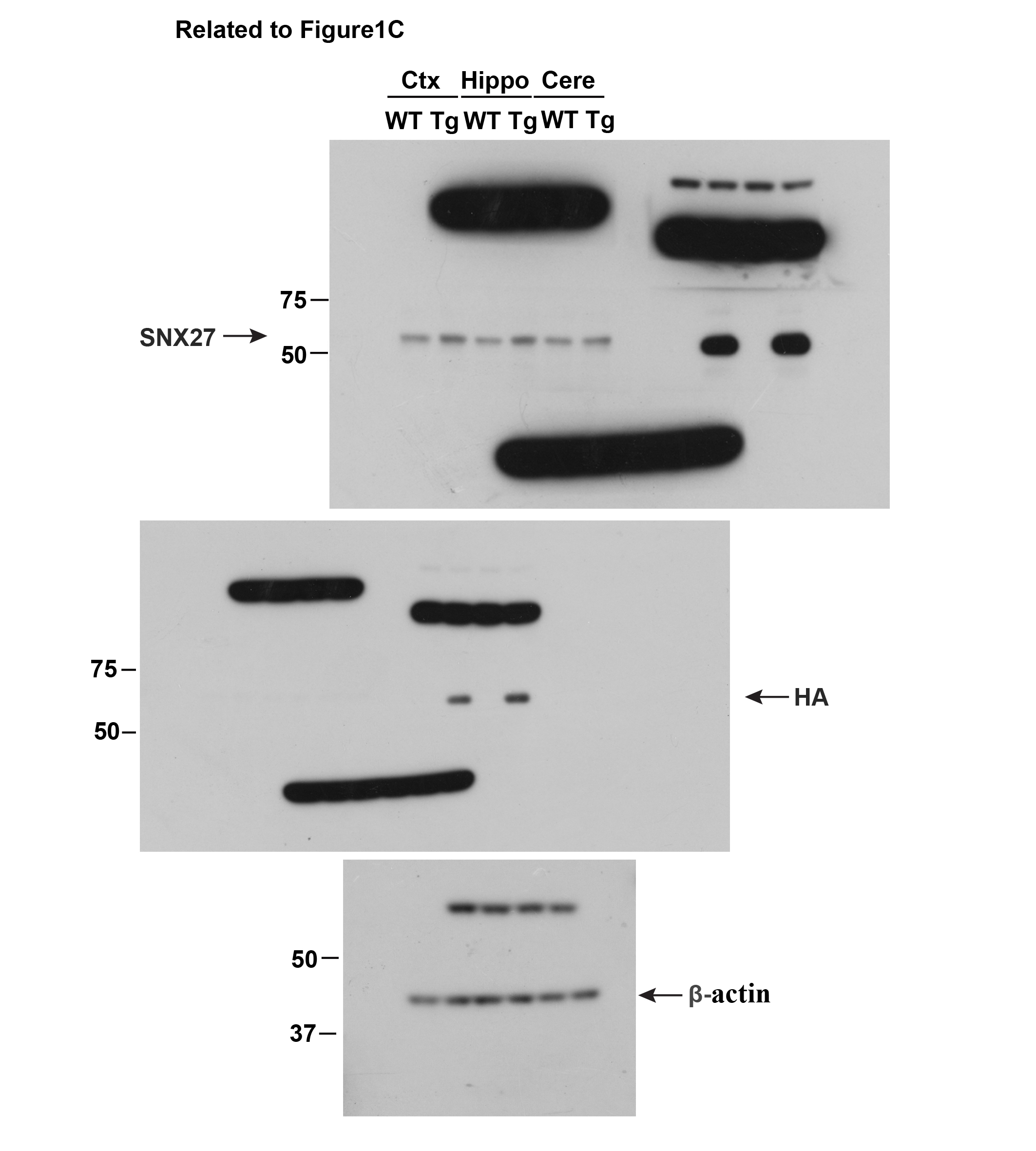

Supplement: Supplementary file 2 [file Data_Sheet_1.ZIP › raw data/Figure 1C.jpg]
